# Association of Clinically Evident Eye Movement Abnormalities With Motor and Cognitive Features in Patients With Motor Neuron Disorders

**DOI:** 10.1212/WNL.0000000000012774

**Published:** 2021-11-02

**Authors:** Barbara Poletti, Federica Solca, Laura Carelli, Alberto Diena, Eleonora Colombo, Silvia Torre, Alessio Maranzano, Lucia Greco, Federica Cozza, Andrea Lizio, Roberta Ferrucci, Floriano Girotti, Federico Verde, Claudia Morelli, Christian Lunetta, Vincenzo Silani, Nicola Ticozzi

**Affiliations:** From the Department of Neurology (B.P., L.C., A.D., E.C., S.T., A.M., F.G., F.V., C.M., V.S., N.T.), Istituto Auxologico Italiano IRCCS; Department of Pathophysiology and Transplantation (F.S., F.V., V.S., N.T.), Dino Ferrari Center and Department of Health Sciences (R.F.), Aldo Ravelli Center for Neurotechnology and Experimental Brain Therapeutics, Universitá degli Studi di Milano; Neuromuscular Omnicenter (L.G., F.C., A.L., C.L.), Fondazione Serena Onlus; Department of Materials Science and COMiB Research Center (F.C.), Università degli Studi di Milano-Bicocca; ASST Santi Paolo e Carlo (R.F.), Neurology Clinic III; and IRCCS Ca Granda Foundation Maggiore Policlinico Hospital (R.F.), Milan, Italy.

## Abstract

**Background and Objectives:**

Although oculomotor abnormalities (OMAs) are not usually considered prominent features of amyotrophic lateral sclerosis (ALS), they may represent potential clinical markers of neurodegeneration, especially when investigated together with cognitive and behavioral alterations. The aim of our study was to identify patterns of clinically evident OMAs in patients with ALS and to correlate such findings with cognitive-behavioral data.

**Methods:**

Three consecutive inpatient cohorts of Italian patients with ALS and controls were retrospectively evaluated to assess the frequency of OMAs and cognitive-behavioral alterations. The ALS population was divided into a discovery cohort and a replication cohort. Controls included a cohort of cognitively impaired individuals and patients with Alzheimer disease (AD). Participants underwent bedside eye movement evaluation to determine the presence and pattern of OMAs. Cognitive assessment was performed with a standard neuropsychological battery (discovery ALS cohort and AD cohort) and the Italian Edinburgh Cognitive and Behavioural ALS Screen (ECAS) (replication ALS cohort).

**Results:**

We recruited 864 individuals with ALS (635 discovery, 229 replication), 798 who were cognitively unimpaired and 171 with AD. OMAs were detected in 10.5% of our ALS cohort vs 1.6% of cognitively unimpaired controls (*p* = 1.2 × 10^−14^) and 11.4% of patients with AD (*p* = NS). The most frequent deficits were smooth pursuit and saccadic abnormalities. OMA frequency was higher in patients with bulbar onset, prominent upper motor neuron signs, and advanced disease stages. Cognitive dysfunction was significantly more frequent in patients with OMAs in both ALS cohorts (*p* = 1.1 × 10^−25^). Furthermore, OMAs significantly correlated with the severity of cognitive impairment and with pathologic scores at the ECAS ALS-specific domains. Last, OMAs could be observed in 35.0% of cognitively impaired patients with ALS vs 11.4% of patients with AD (*p* = 6.4 × 10^−7^), suggesting a possible involvement of frontal oculomotor areas in ALS.

**Conclusion:**

Patients with ALS showed a range of clinically evident OMAs, and these alterations were significantly correlated with cognitive, but not behavioral, changes. OMAs may be a marker of neurodegeneration, and bedside assessment represents a rapid, highly specific tool for detecting cognitive impairment in ALS.

Amyotrophic lateral sclerosis (ALS) is a fatal neurodegenerative disorder affecting upper (UMNs) and lower motor neurons (LMNs). Although ALS has long been considered a pure motor system disorder, it is now accepted that cognitive and behavioral changes suggestive of frontal dysfunction are often part of the clinical syndrome.^1-5^ Several video-oculography studies have also suggested that different types of oculomotor abnormalities (OMAs) may occur in ALS as well.^6,7^ Nevertheless, the frequency of clinically evident OMAs in patients with ALS, as detected by a standard neurologic examination rather than by quantitative video-oculographic measurements, and their association with the underlying motor and cognitive phenotype are still unknown. OMAs in ALS have usually been associated with alterations in the frontal and prefrontal cortex, especially the dorsolateral prefrontal cortex (DLPFC).^8-11^ In this framework, they could represent a potential clinical marker of neurodegeneration beyond the traditional UMN and LMN pathology, providing insight into the pattern and pathogenesis of the disease. Recently, the association between OMAs, assessed by video-oculographic registration, and clinical and neuropsychological performance has been investigated. Significant correlations were found with functional impairment, assessed by the Revised ALS Functional Rating Scale (ALSFRS-R) score, as well as with cognitive scores on the Edinburgh Cognitive and Behavioural ALS Screen (ECAS).^9^

Here, we aim to establish the frequency of clinically evident OMAs in a large cohort of patients with ALS, to identify their patterns, and to correlate such findings with motor and cognitive-behavioral features.

## Methods

### Standard Protocol Approvals, Registrations, and Patient Consents

We received approval for this study from the ethical standard committee on human experimentation of Istituto Auxologico Italiano IRCCS (2013_06_25). Written informed consent for using anonymized clinical data for research purposes was obtained at the time of evaluation from all patients included in the retrospective analysis.

### Patient Cohorts

Inpatient medical records of patients discharged from 2 Italian ALS Centers (Neurology Clinic at Istituto Auxologico Italiano and Neuromuscular Omnicenter Milan Center) between 2008 and 2018 with a primary diagnosis of ALS and other motor neuron disorders (primary lateral sclerosis [PLS] and progressive muscular atrophy [PMA]) were retrospectively evaluated to assess the frequency of OMAs and cognitive or behavioral alterations. The retrospective patient cohort was collected consecutively. Exclusion criteria were as follows: eye diseases preventing bedside evaluation of ocular movements (past eye removal surgery, blindness, orbital myopathies, etc) and end-stage disease with motor and speech disability too severe to perform a bedside evaluation of cognitive functions. The following demographic and clinical information was collected: sex; age at onset; age at diagnosis; site of onset; clinical phenotype (classic, bulbar, respiratory, predominant UMN, flail arm, flail leg, PLS, PMA); ALSFRS-R score at evaluation; progression rate, calculated with the formula (48 − ALSFRS-R score)/disease duration at evaluation expressed in months; time to placement of percutaneous endoscopic gastrostomy or start of noninvasive ventilation; clinical stages according to the King and Milano-Torino (MITOS) staging systems; and presence of *c9orf72* (G_4_C_2_)_n_ repeat expansion. The study population was divided into a discovery cohort, composed of patients with ALS evaluated at Istituto Auxologico Italiano between 2008 and 2013, and a replication cohort recruited at Istituto Auxologico Italiano and Neuromuscular Omnicenter Milan Center between 2013 and 2018.

### Control Cohorts

Two control cohorts were similarly recruited by reviewing inpatient medical records of patients evaluated at the Neurology Clinic of Istituto Auxologico Italiano between 2008 and 2018. The first control group (cognitively unimpaired cohort) includes individuals >30 years at the time of evaluation and discharged with a primary diagnosis of idiopathic headache, syncope, or disorders of the spine, peripheral nervous system, or muscles, as well as those without evidence of neurologic disorders. The second control group (Alzheimer disease [AD] cohort) includes cognitively impaired individuals, as determined by full neuropsychological evaluation, with a CSF biomarker profile compatible with AD. Both control groups were recruited consecutively. Exclusion criteria were the same for both the ALS and control cohorts. Cases and controls were evaluated under similar conditions by the same team of neurologists.

### Ocular Movement Assessment

Bedside eye movement evaluation was routinely performed in patients with ALS and controls by trained neurologists as part of the standard neurologic examination and recorded in a standardized checklist. Briefly, the accuracy of visually guided saccades was evaluated at 20° to 30° gaze excursion for all 4 directions (upward, downward, and horizontal); saccadic movements were considered abnormal when ≥2 corrective saccades were required to reach the target (hypometric saccades). Very slow saccades, as determined qualitatively by the examiner, were also considered to be pathologic. Smooth pursuit was assessed by presenting a horizontally moving target (angular velocity 20°/s–30°/s) and evaluating the appearance of corrective saccades. Gaze limitation was defined as a reduction >50% of the amplitude of saccadic movements in 1 direction. For the purpose of this study, clinical records were independently reviewed to determine the presence of OMAs by 3 neurologists experienced in the field of motor neuron diseases (C.M., C.L., and N.T), who also had a prominent role in the original evaluation of patients with ALS. OMAs were further subdivided into the following 5 categories: saccadic dysfunction, smooth pursuit gain reduction, isolated upward gaze limitation, ocular apraxia, and conjugate gaze palsy. Because of the retrospective design of this study, it was not possible to reliably evaluate the occurrence of other OMAs such as voluntary saccade dysfunction, gaze impersistence, square wave jerks, or convergence abnormalities.

### Cognitive and Behavioral Assessment

For the discovery ALS cohort, bedside mental status evaluation was performed in all cases by trained neurologists and recorded on a standardized checklist. Whenever the suspicion of cognitive impairment or behavioral changes arose, the patient underwent a full neuropsychological evaluation with a standard test battery. On the basis of their performance on specific tests, patients were classified as cognitively normal or presenting ALS-specific cognitive impairment (pathologic scores at Trail Making Test parts B and B-A, Tower of London test, Frontal Assessment Battery, Stroop Color and Word test, Digit Span Backwards test, Token test, Boston Naming test, Verbal Fluency test, or the Sartori Naming test), ALS-nonspecific cognitive impairment (pathologic scores on the Mini-Mental State Examination, Attentive Matrices test, Trail Making Test Part A, Digit Span Forward, Raven Progressive Matrices, Clock Drawing test, Paired-Associate Learning test, Short Story Recall, Spatial Span test, Constructional Apraxia test, Street Completion test), or both. Conversely, in the replication cohort, all patients, independently from the clinical suspicion, were systematically assessed for the presence of both cognitive and behavioral alterations using the ECAS–Italian version^12^ and subsequently classified according to the Strong revised criteria^3^ into the following categories: ALS with normal cognition, ALS with behavioral impairment (ALSbi), ALS with cognitive impairment (ALSci), ALS with cognitive and behavioral impairment (ALScbi), and ALS with concurrent frontotemporal dementia (ALS-FTD). With regard to the cognitive assessment, the following variables were considered: ECAS total score, ALS-specific score, ALS-nonspecific score, and individual domain subscores (language, verbal fluency, executive, memory, visuospatial). Each score was classified as normal vs pathologic according to validated cutoff values.^12^ For behavioral evaluation, the following variables were considered: ECAS behavior screen score, presence of individual symptoms (disinhibition, apathy/inertia, loss of sympathy/empathy, perseveration, hyperorality), and ALS psychosis screen score.

For the unimpaired control cohort, absence of cognitive impairment was determined at the bedside clinical evaluation according to the above-mentioned standardized checklist. Patients with AD underwent a full neuropsychological examination with the standard test battery already described.

### Statistical Analysis

The Pearson χ^2^ test was used to assess differences between groups for categorical variables. The Fisher exact test was preferred when testing small samples. The Mann-Whitney *U* test was used for continuous data. Kaplan-Meier curves followed by the log-rank test were used to evaluate survival of different groups. Values of *p* < 0.05 were considered significant after Bonferroni correction for multiple testing when appropriate. Due to the small percentage of inpatient records missing bedside eye movement assessment, listwise deletion was chosen to handle missing data when comparing OMAs in cases vs controls and in cognitively impaired vs unimpaired patients with ALS. Pairwise deletion was used to handle missing data for correlations with other phenotypic traits. Statistical analysis was performed with IBM Statistical Package for Social Science (SPSS) version 26 (Armonk, NY).

### Data Availability

Anonymized data of cases and controls, as well as the checklists used for bedside eye movement and cognitive evaluation, are archived on Zenodo (https://doi.org/10.5281/zenodo.4573109) and will be made available and shared by reasonable request from any qualified investigator.

## Results

**Figure 1:**
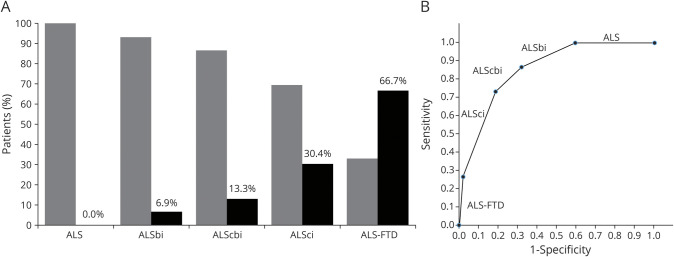
OMAs Distribution According to Presence of Cognitive or Behavioral Alterations in the Replication Cohort (A) and ROC Curve for OMAs According to the Revised Strong Criteria (B) Black bars indicate patients with ocular movement abnormalities (OMAs); gray bars indicate patients with normal ocular movements. Bonferroni-adjusted *p* values for pairwise comparisons between different Edinburgh Cognitive and Behavioural ALS Screen categories: amyotrophic lateral sclerosis (ALS) vs ALS with cognitive impairment (ALSci), ALS vs ALS with frontotemporal dementia (ALS-FTD), ALS with behavioral impairment (ALSbi) vs ALS-FTD (*p* < 0.0001); ALS vs ALS with cognitive and behavioral impairment (ALScbi) (*p* = 0.049); ALSbi vs ALSci (*p* = 0.032); ALScbi vs ALS-FTD (*p* = 0.013); ALSci vs ALS-FTD (*p* = 0.004); and ALS vs ALSbi, ALSbi vs ALScbi, and ALScbi vs ALSci (*p* = NS). Area under the receiver operating characteristic curve is 0.92 (95% confidence interval 0.89–0.96).

To determine the frequency of OMAs in motor neuron disease, we retrospectively analyzed the inpatient clinical records of 2 independent cohorts of 635 and 229 patients with ALS. The demographic and clinical characteristics of both groups are summarized in Table 1. The ocular movement evaluation checklist was filled out thoroughly in 624 of 635 (98.3%) and 225 of 229 (98.2%) medical records, respectively. OMAs could be detected at bedside neurologic examination in 59 of 624 (9.5%) individuals belonging to the discovery cohort and in 30 of 225 (13.3%) cases in the replication cohort for a global frequency of 10.5% in our ALS population. The frequency and distribution of OMA types were not statistically different between the 2 cohorts. In particular, we observed saccadic dysfunctions in 17 (2.0%), smooth pursuit abnormalities in 59 (6.9%), upward gaze limitation in 33 (3.9%), ocular apraxia in 7 (0.8%), and conjugate gaze palsy in 7 (0.8%) cases (Table 2). To rule out the possibility of a nonspecific finding, given the association between OMAs and increasing age, we retrospectively analyzed the inpatient medical records of 798 cognitively unimpaired, age-matched controls (54 with primary headaches, 54 with syncope, 176 with spine diseases or myelopathies, 204 with neuropathies, 69 with myopathies, 17 with other neurologic conditions, 224 without evidence of CNS or peripheral nervous system disorders). The ocular movement evaluation checklist was filled out thoroughly in 789 of 798 (98.9%) medical records of the control cohort. In our population, OMAs occur significantly more frequently in patients with ALS compared to controls (89 of 849 vs 13 of 789; 10.5% vs 1.6%; odds ratio [OR] 7.0 [95% confidence interval (CI) 3.9–12.6]; *p* = 1.2 × 10^−14^). We also analyzed a second control group composed of 171 patients with cognitive impairment due to AD pathology. The ocular movement checklist was filled out thoroughly in 167 (97.7%) medical records. We observed a higher occurrence of OMAs in patients with AD compared to cognitively unimpaired individuals (19 of 167 vs 13 of 789; 11.4% vs 1.6%; OR 7.7 [95% CI 3.7–15.9]; *p* = 6.9 × 10^−8^), while the frequency was similar to that of the ALS cohort (19 of 167 vs 89 of 849; 11.4% vs 10.5%; *p* = NS). With regard to clinical phenotype, we observed an increased frequency of OMAs in bulbar-onset patients compared to those with spinal onset (16.4% vs 8.8%; OR 2.0 [95% CI 1.3–3.2]; *p* = 2.5 × 10^−3^). Age at onset was also higher in the OMA group compared with patients with normal eye movements (65.7 vs 58.6 years; *p* = 4.9 × 10^−8^). In addition, OMAs often appear to be associated with phenotypes characterized by prominent UMN involvement (PLS and predominant UMN) compared to classic ALS and predominantly LMN diseases (flail arm, flail leg, PMA) (21.6% vs 7.7% vs 2.1%; *p* = 3.0 × 10^−6^) (Table 3). OMAs also seem to correlate with the severity of disease spreading, with higher frequencies in patients with King stages 3 and 4 compared to 1 and 2 (13.1% vs 5.3%, OR 5.7 [95% CI 1.4–5.1]; *p* = 1.5 × 10^−3^). No association could be observed with MITOS stages, ALSFRS-R total score, and subdomain score. Given the different methods used for cognitive evaluation (i.e., standard neuropsychological battery for selected patients for whom the suspicion of cognitive impairment arose at bedside evaluation for the discovery cohort vs systematic screening using ECAS for the replication cohort), we observed significant differences in the frequency of cognitive impairment in the 2 populations. In particular, within the discovery cohort, we identified 55 (8.8%) patients with ALS with concurrent signs of cognitive dysfunction. Of these, 8 (1.3%) had an ALS-specific type of impairment, 12 (2.0%) had an ALS-nonspecific type, while 32 (5.2%) showed both. Conversely, in the replication cohort, 88 (39.1%) patients displayed signs of cognitive impairment (46 [20.4%] ALSci, 30 [13.3%] ALScbi, 12 [5.3%] ALS-FTD), 58 (25.6%) had signs of behavioral dysfunction only, while 79 (35.1%) had normal cognition. We observed that cognitive dysfunction occurred with a significantly higher frequency in patients with ALS with OMAs compared to individuals with normal oculomotor function in both the discovery (24 of 59 vs 31 of 565; 40.7% vs 5.5%; OR 11.8 [95% CI 6.3–22.2]; *p* = 1.2 × 10^−19^) and replication (26 of 30 vs 62 of 195; 86.7% vs 31.8%; OR 4.0 [95% CI 1.9–8.2]; *p* = 9.8 × 10^−9^) cohorts, as well as in both cohorts combined (50 of 89 vs 93 of 760; 56.2% vs 12.2%; OR 9.2 [95% CI 5.7–17.7]; *p* = 1.1 × 10^−25^) (Table 4). Although the overall frequency was similar in the ALS and AD cohorts, it is worth noting that OMAs occur significantly more often in cognitively impaired individuals with ALS compared to patients with AD (50 of 143 vs 19 of 167; 35.0% vs 11.4%; OR 4.2 [95% CI 2.3–7.5]; *p* = 6.4 × 10^−7^). OMAs appear to be a highly specific although less sensitive proxy for the presence of cognitive impairment in ALS (specificity 94.5% [95% CI 92.5%–96.0%]; sensitivity 35.0% [95% CI 27.2%–43.3%]; positive likelihood ratio 6.3 [95% CI 4.3–9.2]; negative likelihood ratio 0.7 [95% CI 0.6–0.8]; positive predictive value 56.2% [95% CI 46.7%–65.2%], negative predictive value 87.8% [95% CI 86.4%–89.0%]) with a global accuracy of 84.5% (95% CI 81.8%–86.8%). In particular, in the combined cohort, the association with cognitive impairment could be observed for all types of OMAs, namely saccadic dysfunctions (13 of 17 vs 130 of 702; 60.0% vs 8.0%; OR 17.6 [95% CI 5.6–54.7]; *p* = 3.2 × 10^−11^), smooth pursuit abnormalities (30 of 59 vs 113 of 790; 28.9% vs 7.5%; OR 6.2 [95% CI 3.6–10.7]; *p* = 4.7 × 10^−13^), upward gaze limitation (20 of 33 vs 123 of 816; 53.8% vs 6.9%; OR 8.7 [95% CI 4.2–17.9]; *p* = 7.3 × 10^−13^), ocular apraxia (5 of 7 vs 138 of 842; 66.7% vs 8.3%; OR 12.6 [95% CI 2.5–66.4]; *p* = 1.1 × 10^−4^), and conjugate gaze palsy (6 of 7 vs 137 of 842; 75.0% vs 8.4%; OR 30.9 [95% CI 3.7–258.5]; *p* = 1.0 × 10^−6^) (Table 4). Furthermore, we detected a significant association between oculomotor dysfunction and the severity of cognitive impairment according to the revised Strong criteria. In fact, in the replication cohort, OMAs were observed in 4 of 58 (6.9%) of ALSbi, 4 of 30 (13.3%) of ALScbi, 14 of 46 (30.4%) of ALSci, and 8 of 12 (66.7%) of ALS-FTD cases, while they could not be detected in patients with normal cognition (Figure 1, A and B). With regard to the association with specific cognitive profiles, within the discovery cohort, we found a higher frequency of OMAs in cases with all types of cognitive impairment compared with unimpaired patients. This difference was particularly relevant for individuals displaying ALS-specific (OR 17.7 [95% CI 4.1–76.2]; *p* = 1.8 × 10^−7^) or both specific and nonspecific (OR 9.7 [95% CI 4.5–20.9]; *p* = 5.5 × 10^−12^) cognitive impairment, while it was less evident for patients with pathologic scores at ALS-nonspecific tests only (OR 5.1 (95% CI 1.5–17.7]; *p* = 4.0 × 10^−3^). Conversely, in the replication cohort, we observed that OMAs were strongly associated with pathologic scores in the language (OR 7.0 [95% CI 2.9–16.7]; *p* = 2.0 × 10^−5^), executive (OR 5.6 [95% CI 2.3–13.2]; *p* = 9.1 × 10^−5^), and visuospatial (OR 4.8 [95% CI 1.8–12.8]; *p* = 3.0 × 10^−3^) ECAS domains and marginally associated with the verbal fluency domain (OR 2.5 [95% CI 1.0–5.9]; *p* = 0.045), while no association was detected with pathologic memory scores. These findings appear to be driven mostly by saccadic dysfunction and smooth pursuit abnormalities, while no association was detected for upward gaze limitation (Table 5). Conversely, the above-mentioned increased frequency of OMAs in patients with ALSbi notwithstanding, we did not observe any association with the burden of behavioral changes, determined as the number of symptoms at the ECAS Behavior Screen (Table 5), as well as with the presence of individual behavioral symptoms and with the ALS Psychosis Screen (data not shown). Last, the presence of oculomotor dysfunction in patients with ALS is not associated with shorter survival time, with an earlier initiation of noninvasive ventilation or placement of percutaneous gastrostomy, or with the presence of the (G_4_C_2_)_n_ repeat expansion in the *c9orf72* gene (Table 3).

**Table 1:**
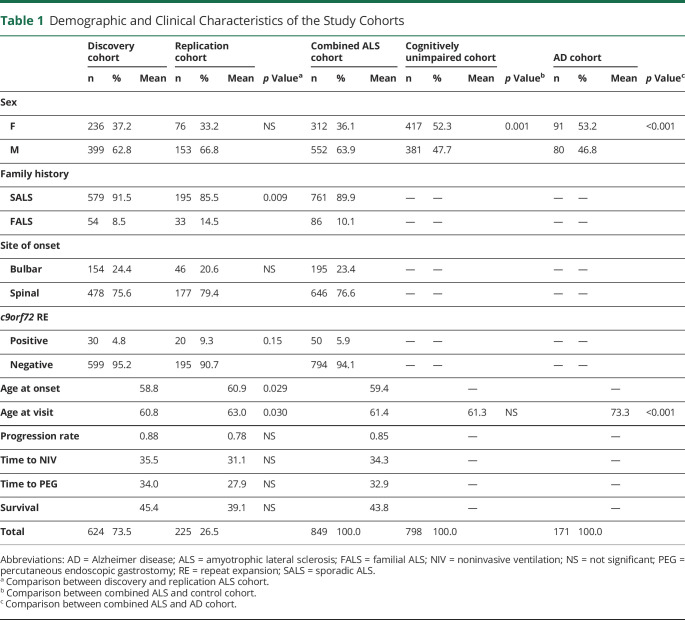
Demographic and Clinical Characteristics of the Study Cohorts

**Table 2:**
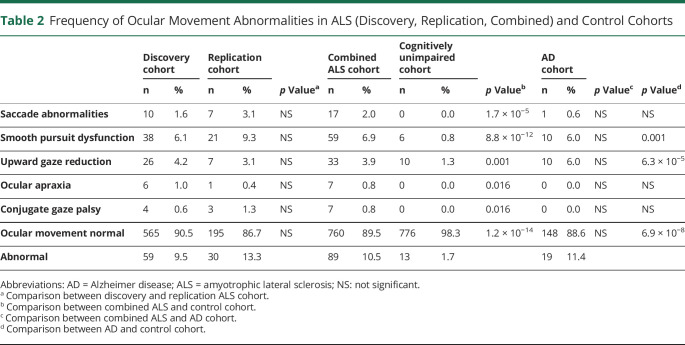
Frequency of Ocular Movement Abnormalities in ALS (Discovery, Replication, Combined) and Control Cohorts

**Table 3:**
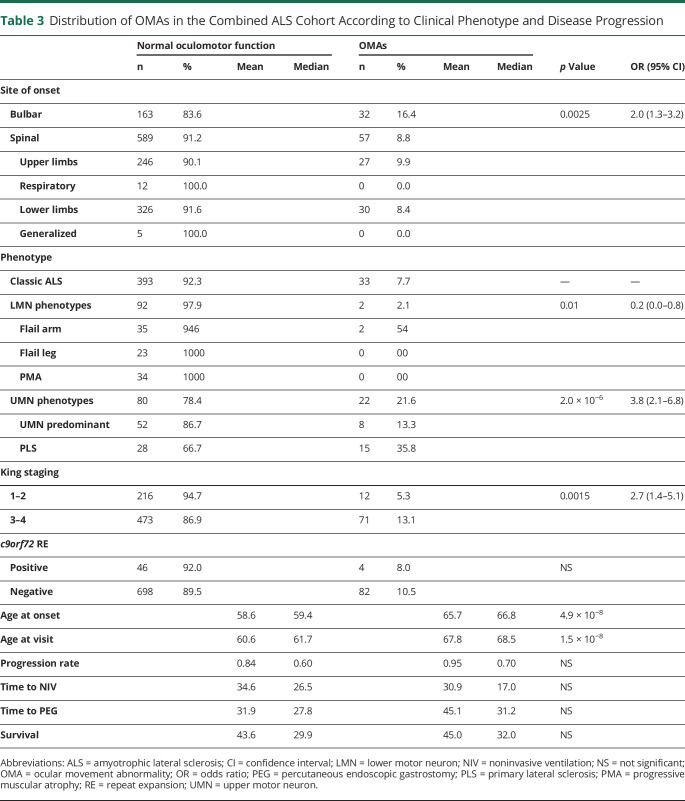
Distribution of OMAs in the Combined ALS Cohort According to Clinical Phenotype and Disease Progression

**Table 4:**
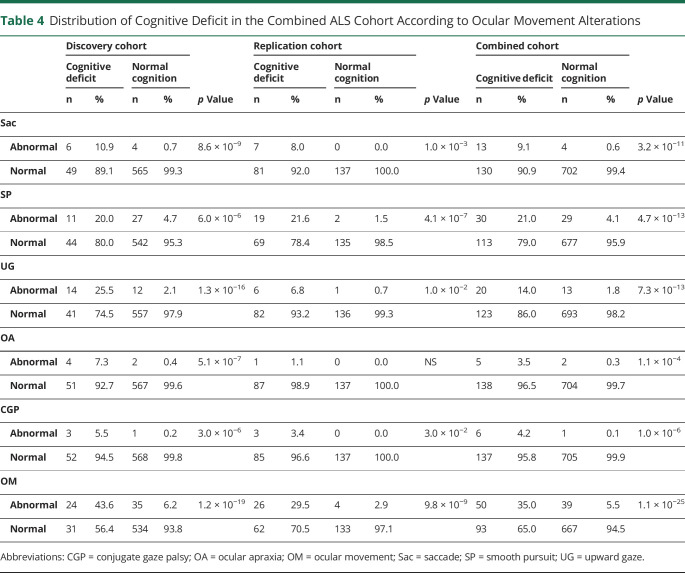
Distribution of Cognitive Deficit in the Combined ALS Cohort According to Ocular Movement Alterations

**Table 5:**
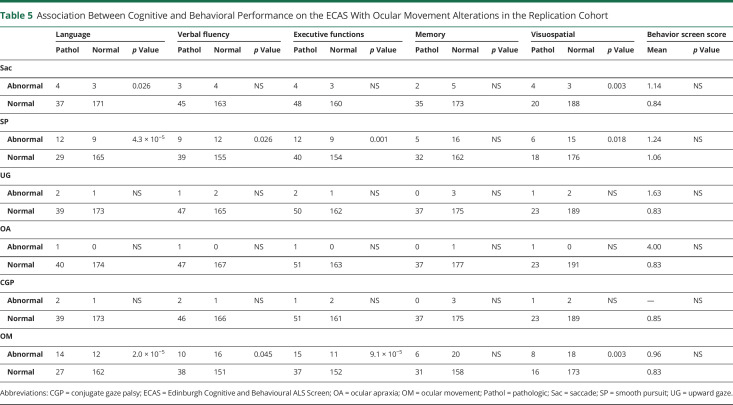
Association Between Cognitive and Behavioral Performance on the ECAS With Ocular Movement Alterations in the Replication Cohort

## Discussion

ALS has traditionally been considered a disease exclusively affecting motor neurons and sparing ocular function. Even if it is now increasingly recognized that a proportion of patients with ALS display extramotor features such as cognitive or behavioral disturbances and extrapyramidal signs, the occurrence and pattern of oculomotor dysfunction in ALS are still debated. For many years, OMAs have been described only in patients with atypical ALS–progressive supranuclear palsy phenotypes or undergoing long-term mechanical ventilation.^13,14^ More recently, an increasing number of studies have detected a broad range of OMAs in patients with ALS^15^ such as ocular fixation instability,^8,16^ saccadic impairment,^8,9,17-23^ increased error rate in antisaccade performance,^10,11,23,24^ defective smooth pursuits,^9,17,20-22,25-27^ gaze palsy,^9,28^ ophthalmoplegia,^14,29^ nystagmus,^17,30^ eyelid opening apraxia,^27^ and abnormal Bell phenomenon.^31^ The majority of these studies, however, quantitatively assessed OMAs by video-oculography recording in relatively small ALS cohorts. Thus, it is still unknown how often clinically evident OMAs, as determined by a standard bedside neurologic examination, occur in patients with ALS.

By examining 2 large independent cohorts of Italian patients with ALS, our study suggests that clinically evident OMAs represent a relatively common feature. In particular, they occur in 10.5% of our global ALS population, with smooth pursuit dysfunction, upward gaze reduction, and saccade abnormalities as the most evident alterations recorded. This frequency is >6-fold higher than that observed in age-matched nonneurologic controls or in neurologic patients without CNS disorders, suggesting that clinically detectable OMAs are part of the phenotype in a significant portion of patients with ALS. The presence of OMAs appears to correlate with disease severity, with higher frequencies in patients with more advanced King stages; however, no association has been observed with ALS-MITOS stages or with ALSFRS-R total and subdomain scores, suggesting that OMAs are associated with anatomic disease spread but not with increasing disability. As previously reported, the presence of OMAs is associated with bulbar onset.^17^ In addition, we demonstrated that OMAs occur more frequently in phenotypes characterized by prominent UMN signs compared to classic ALS and predominantly LMN diseases. In both the discovery and replication cohorts, as well as in the overall population, we found a highly significant association between all OMA types and impaired cognition, as well as with the degree of severity and specific profiles of cognitive dysfunction observed. With regard to the former point, in the replication cohort, the frequency and complexity (i.e., ocular apraxia and conjugate gaze palsy) of OMAs increased with the severity of cognitive impairment; they occurred more often in patients diagnosed with ALS-FTD, followed by those with ALSci and ALScbi, compared to those with a normal cognitive profile. Unexpectedly, patients diagnosed with ALScbi displayed a lower frequency of OMAs compared to those diagnosed with ALSci, a finding that could be explained by the relatively small number of cases of ALScbi in the replication cohort. Two patients with ALS-FTD and conjugate gaze palsy also displayed extrapyramidal signs compatible with an ALS–progressive supranuclear palsy phenotype. Concerning the qualitative nature of the impairment, we observed an association between OMAs and cognitive dysfunctions characteristic of ALS. In fact, in the discovery cohort, a higher frequency of OMAs was found in patients showing ALS-specific cognitive impairment compared with cognitively unimpaired patients, while this association was weaker for patients with pathologic scores on ALS-nonspecific tests only. Because the ALS-specific impairment reflects a poor performance on tests sensitive to cognitive functions often affected in ALS (i.e., frontal lobe–dependent tasks, executive functions, language and verbal fluency tests), these results are in line with previous findings showing significant correlations between oculomotor parameters and frontal lobe dependent tasks.^9,10,20,32^ The results obtained in the discovery cohort have been confirmed in the replication cohort, in which OMAs, in particular saccadic dysfunction and smooth pursuit gain reduction, strongly correlated with ECAS ALS-specific subscores, particularly the language and executive domains and, to a lesser extent, the fluency domain. An association was also observed with the visuospatial domain, but it must be noted that patients with impaired performance on this domain also scored the lowest on the other ECAS subtests. We can thus hypothesize that this finding is nonspecific and likely due to a more pervasive cognitive impairment of these patients.

**Figure 2:**
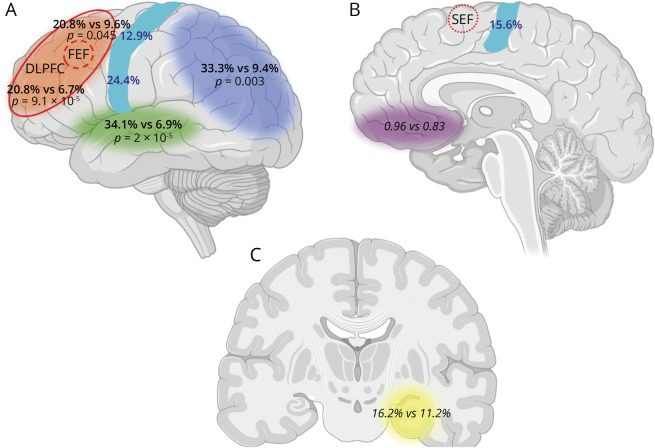
Association of Oculomotor Abnormalities With Cognitive-Behavioral Alterations and Site of Onset in Patients With ALS From the Replication Cohort Cerebral areas most prominently involved in the different cognitive functions investigated by neuropsychological evaluation were language (green, A), verbal fluency and executive functions (orange, A), memory (yellow, C), and visuospatial functions (blue, A), as well as the primary motor cortex (light blue strip, A and B). (A) Lateral aspect of brain. (B) Medial aspect. (C) Coronal section at the level of the hippocampi. Verbal fluency and executive functions are considered together from a topographic standpoint because they are both localized in the dorsolateral prefrontal cortex (DLPFC). In addition, the orbitomedial prefrontal cortex is highlighted in purple as the most relevant cortical area for behavior. Within each cortical area and corresponding cognitive domain, the percentage frequencies of oculomotor abnormalities among patients with and without impairments, respectively, in the relative domains are reported (black). Within the orange area, the percentages written above pertain to verbal fluency, while those written below pertain to executive functions. For behavioral alterations, the behavior screen scores of patients with vs without oculomotor abnormalities are reported. Comparisons resulting in statistically significant differences are in bold, and the *p* value is shown. In the primary motor cortex, the percentage frequencies of oculomotor abnormalities in patients with disease onset in different body segments are reported in dark blue, according to the somatotopic representation of motor control within the motor strip (bulbar region and upper limbs: lower and upper parts of the motor strip in the lateral aspect of the brain, respectively, A; lower limbs: part of the motor strip lying in the upper part of the medial aspect of the hemisphere, B). In addition to the DLPFC (delimited by the continuous red line), the frontal eye field (FEF; located within the DLPFC itself) and the supplemental eye field (SEF) are depicted (areas within the dashed red line and within the pointed red line, respectively). Image created with BioRender.

Overall, our study supports the hypothesis that the appearance in ALS of OMAs, in particular increased latency and reduced amplitude of saccadic movements, can be attributed mostly to a dysfunction of the DLPFC and the frontal (FEF) and supplemental (SEF) eye fields.^6^ Thus, OMAs could represent an appealing proxy to monitor the disease progression within the CNS from the primary motor cortex toward the prefrontal cortex, with the consequent appearance of language and executive dysfunction, a phenomenon that in our cohort is particularly evident in patients with ALS with prominent UMN involvement. The observation that OMAs occur significantly more frequently in cognitively impaired patients with ALS vs patients with AD further strengthens the hypothesis of a neuropathologic involvement of frontal oculomotor areas (FEF, SEF, DLPFC) in ALS compared to AD, in which predominant temporo-parieto-occipital dysfunctions would be expected (Figure 2).

Smooth pursuit impairment is a less specific sign of frontal dysfunction, being associated to lesions within the FEF and SEF but also within extrapyramidal and cerebellar pathways. In fact, a recent video-oculography study has suggested that OMA complexity in ALS increases with disease severity, starting from deficits in executive eye control movements and progressing to brainstem and precerebellar/pontine circuit dysfunction,^9^ being consistent with the Braak staging of phosphorylated TAR DNA-binding protein 43 pathology.^33^

Conversely, we could not detect any association between OMAs and the presence of behavioral impairment in patients with ALS in the replication cohort, determined as the number of symptoms registered on the ECAS Behavior and ALS Psychosis screen. To date, few studies have investigated such a relationship. One study did not find any correlation between OMAs and measurement of behavioral alteration through the Frontal Behavioral Inventory.^11^ The ECAS has recently been used to investigate the cognitive profile of patients with ALS with OMAs, but the scores obtained with the ECAS Behavior Interview were not reported.^9,34^ We hypothesize that the lack of association with OMAs can be explained by the prominent involvement of the orbitomedial prefrontal cortex compared to the DLPFC in patients with ALSbi.^35^ It must be remarked, however, that systematic behavioral data are available for the replication cohort only. Hence, the lack of association between OMAs and behavioral impairment in our study may also be due to the relatively smaller sample size.

This study has limitations. One limitation is the above-mentioned nonhomogeneity in the neuropsychological data between the 2 ALS cohorts, with the discovery cohort being assessed with a standard cognitive test battery only when the suspicion of cognitive or behavioral impairment arose and the replication cohort systematically assessed with the ECAS. Therefore, although the frequency of cognitive or behavioral impairment in the replication cohort is consistent with the literature,^36^ the percentage of patients with some degree of alteration is significantly lower in the discovery cohort. The low detection rate of cognitive impairment in the whole cohort could thus be explained by the lack of a systematic neuropsychological screening in patients belonging to the discovery cohort because data collection started well before ECAS development and implementation in the clinical setting. Moreover, bedside ocular movement assessment may have some biases as well. A possible confounding factor is the known association of certain types of OMAs such as upward gaze limitation with increasing age.^37^ Although this association was observed in both our ALS and control cohorts, it must be noted that OMAs occurred significantly more frequently in cases. The qualitative nature of bedside eye movement assessment could also have led to an underestimation of the less severe types of OMAs in our patients, especially because they are not a core clinical feature of ALS, thus resulting in a lower sensitivity. For the same reason, another valid concern is the possible lack of completeness of the section concerning eye movement evaluation within medical records and that the bedside eye movement evaluation was carried out by different clinicians over the years. To overcome these possible limitations, we decided to study 2 retrospective inpatient ALS cohorts in which the bedside eye movement evaluation was performed by the same teams of trained neurologists in all cases and the accuracy and completeness of data collected are higher than in an outpatient cohort, as demonstrated by the small percentage of missing data. In fact, although no interrater reliability statistics could be performed, it should be observed that the OMA detection rate in our sample did not change over the years or between the discovery and replication cohorts, suggesting that bedside eye movement assessment is a fairly accurate and reliable clinical tool. Last, any retrospective study such as ours inevitably lacks a priori standardized clinical evaluation protocols. To minimize this possible bias and to guarantee that all cases and controls were evaluated with the same ocular movement and cognitive assessment checklists, we consecutively included every individual meeting the inclusion and exclusion criteria admitted to our institutions between 2008 and 2018.

Our study demonstrates that clinically evident OMAs are a highly specific although less sensitive proxy for the presence of cognitive impairment in ALS. Bedside oculomotor evaluation thus represents an easy and inexpensive clinical tool allowing rapid identification of patients with ALS with unimpaired cognition and the selection of possibly impaired patients for more in-depth neuropsychological assessment. Longitudinal studies on independent prospective cohorts will be needed to determine the accuracy of bedside OMA detection in monitoring the clinical and cognitive progression in patients with ALS as well.

## Glossary

AD: Alzheimer disease
ALS: amyotrophic lateral sclerosis
ALSbi: ALS with behavioral impairment
ALScbi: ALS with cognitive and behavioral impairment
ALSci: ALS with cognitive impairment
ALSFRS-R: Revised ALS Functional Rating Scale
ALS-FTD: ALS with frontotemporal dementia
CI: confidence interval
DLPFC: dorsolateral prefrontal cortex
ECAS: Edinburgh Cognitive and Behavioural ALS Screen
FEF: frontal eye field
LMN: lower motor neuron
MITOS: Milano-Torino staging
OMA: oculomotor abnormality
OR: odds ratio
PLS: primary lateral sclerosis
PMA: progressive muscular atrophy
SEF: supplemental eye field
UMN: upper motor neuron
